# Supplementary material for: Assessment of a decentralization model in improving treatment and care of visceral leishmaniasis in Turkana County, Kenya: A mixed method study
Source: PLoS One. 2025 May 20;20(5):e0323990. doi: 10.1371/journal.pone.0323990 (PMC12091879; doi:10.1371/journal.pone.0323990)
Supplement: Attachment — Submitted filename: Response to Reviewers.docx [file pone.0323990.s002.docx]

PONE-D-24-30004

Assessment of a decentralization model in improving treatment and care of visceral leishmaniasis in Turkana County, Kenya: A mixed method study

PLOS ONE

Dear Dr. Okoyo,

Thank you for submitting your manuscript to PLOS ONE. After careful consideration, we feel that it has merit but does not fully meet PLOS ONE’s publication criteria as it currently stands. Therefore, we invite you to submit a revised version of the manuscript that addresses the points raised during the review process.

Please submit your revised manuscript by Nov 29 2024 11:59PM. If you will need more time than this to complete your revisions, please reply to this message or contact the journal office at <a href="mailto:plosone@plos.org">plosone@plos.org. When you're ready to submit your revision, log on to https://www.editorialmanager.com/pone/ and select the 'Submissions Needing Revision' folder to locate your manuscript file.

Please include the following items when submitting your revised manuscript:

- A rebuttal letter that responds to each point raised by the academic editor and reviewer(s). You should upload this letter as a separate file labeled 'Response to Reviewers'.
- A marked-up copy of your manuscript that highlights changes made to the original version. You should upload this as a separate file labeled 'Revised Manuscript with Track Changes'.
- An unmarked version of your revised paper without tracked changes. You should upload this as a separate file labeled 'Manuscript'.

If you would like to make changes to your financial disclosure, please include your updated statement in your cover letter. Guidelines for resubmitting your figure files are available below the reviewer comments at the end of this letter.

If applicable, we recommend that you deposit your laboratory protocols in protocols.io to enhance the reproducibility of your results. Protocols.io assigns your protocol its own identifier (DOI) so that it can be cited independently in the future. For instructions see: <https://journals.plos.org/plosone/s/submission-guidelines#loc-laboratory-protocols>. Additionally, PLOS ONE offers an option for publishing peer-reviewed Lab Protocol articles, which describe protocols hosted on protocols.io. Read more information on sharing protocols at <https://plos.org/protocols?utm_medium=editorial-email&utm_source=authorletters&utm_campaign=protocols>.

We look forward to receiving your revised manuscript.

Kind regards,

Mehdi Bamorovat
Academic Editor
PLOS ONE

Journal Requirements:

When submitting your revision, we need you to address these additional requirements.

1. Please ensure that your manuscript meets PLOS ONE's style requirements, including those for file naming. The PLOS ONE style templates can be found at

<https://journals.plos.org/plosone/s/file?id=wjVg/PLOSOne_formatting_sample_main_body.pdf> and

<https://journals.plos.org/plosone/s/file?id=ba62/PLOSOne_formatting_sample_title_authors_affiliations.pdf>

>> We have checked and ensured that our manuscript meets PLOS ONE’s style requirements.

2. We note that Figure 1 in your submission contain [map/satellite] images which may be copyrighted. All PLOS content is published under the Creative Commons Attribution License (CC BY 4.0), which means that the manuscript, images, and Supporting Information files will be freely available online, and any third party is permitted to access, download, copy, distribute, and use these materials in any way, even commercially, with proper attribution. For these reasons, we cannot publish previously copyrighted maps or satellite images created using proprietary data, such as Google software (Google Maps, Street View, and Earth). For more information, see our copyright guidelines: <http://journals.plos.org/plosone/s/licenses-and-copyright>.

We require you to either (1) present written permission from the copyright holder to publish these figures specifically under the CC BY 4.0 license, or (2) remove the figures from your submission:

     a. You may seek permission from the original copyright holder of Figure 1 to publish the content specifically under the CC BY 4.0 license.

We recommend that you contact the original copyright holder with the Content Permission Form (http://journals.plos.org/plosone/s/file?id=7c09/content-permission-form.pdf) and the following text:

“I request permission for the open-access journal PLOS ONE to publish XXX under the Creative Commons Attribution License (CCAL) CC BY 4.0 (http://creativecommons.org/licenses/by/4.0/). Please be aware that this license allows unrestricted use and distribution, even commercially, by third parties. Please reply and provide explicit written permission to publish XXX under a CC BY license and complete the attached form.”

Please upload the completed Content Permission Form or other proof of granted permissions as an "Other" file with your submission.

In the figure caption of the copyrighted figure, please include the following text: “Reprinted from [ref] under a CC BY license, with permission from [name of publisher], original copyright [original copyright year].”

   b. If you are unable to obtain permission from the original copyright holder to publish these figures under the CC BY 4.0 license or if the copyright holder’s requirements are incompatible with the CC BY 4.0 license, please either i) remove the figure or ii) supply a replacement figure that complies with the CC BY 4.0 license. Please check copyright information on all replacement figures and update the figure caption with source information. If applicable, please specify in the figure caption text when a figure is similar but not identical to the original image and is therefore for illustrative purposes only.

The following resources for replacing copyrighted map figures may be helpful:

USGS National Map Viewer (public domain): <http://viewer.nationalmap.gov/viewer/>

The Gateway to Astronaut Photography of Earth (public domain): <http://eol.jsc.nasa.gov/sseop/clickmap/>

Maps at the CIA (public domain): https://www.cia.gov/library/publications/the-world-factbook/index.html and <https://www.cia.gov/library/publications/cia-maps-publications/index.html>

NASA Earth Observatory (public domain): <http://earthobservatory.nasa.gov/>

Landsat: <http://landsat.visibleearth.nasa.gov/>

USGS EROS (Earth Resources Observatory and Science (EROS) Center) (public domain): [http://eros.usgs.gov/#](http://eros.usgs.gov/)

>> The original map in Figure 1 has been deleted and another one provided which is now showing the selected study health facilities. The map was created using ArcGIS Desktop version 10.2.2 software (Environmental Systems Research Institute Inc., Redlands, CA, USA). The base layer of the map was obtained from Environmental Systems Research Inc., (ESRI) (<https://www.esri.com/en-us/arcgis/products/arcgis-online/features/make-maps>).

3. In this instance it seems there may be acceptable restrictions in place that prevent the public sharing of your minimal data. However, in line with our goal of ensuring long-term data availability to all interested researchers, PLOS’ Data Policy states that authors cannot be the sole named individuals responsible for ensuring data access (<http://journals.plos.org/plosone/s/data-availability#loc-acceptable-data-sharing-methods>).

Data requests to a non-author institutional point of contact, such as a data access or ethics committee, helps guarantee long term stability and availability of data. Providing interested researchers with a durable point of contact ensures data will be accessible even if an author changes email addresses, institutions, or becomes unavailable to answer requests.

Before we proceed with your manuscript, please also provide non-author contact information (phone/email/hyperlink) for a data access committee, ethics committee, or other institutional body to which data requests may be sent. If no institutional body is available to respond to requests for your minimal data, please consider if there any institutional representatives who did not collaborate in the study, and are not listed as authors on the manuscript, who would be able to hold the data and respond to external requests for data access? If so, please provide their contact information (i.e., email address). Please also provide details on how you will ensure persistent or long-term data storage and availability.

>> We have moved the minimal anonymized data to the GitHub public repository link, see (<https://github.com/mancollo/VL-Decentralisation-Paper>).

[Note: HTML markup is below. Please do not edit.]

Reviewers' comments:

Reviewer's Responses to Questions

**Comments to the Author**

1. Is the manuscript technically sound, and do the data support the conclusions?

The manuscript must describe a technically sound piece of scientific research with data that supports the conclusions. Experiments must have been conducted rigorously, with appropriate controls, replication, and sample sizes. The conclusions must be drawn appropriately based on the data presented.

Reviewer #1: Yes

Reviewer #2: Yes

2. Has the statistical analysis been performed appropriately and rigorously?

Reviewer #1: I Don't Know

Reviewer #2: No

3. Have the authors made all data underlying the findings in their manuscript fully available?

The [PLOS Data policy](http://www.plosone.org/static/policies.action#sharing) requires authors to make all data underlying the findings described in their manuscript fully available without restriction, with rare exception (please refer to the Data Availability Statement in the manuscript PDF file). The data should be provided as part of the manuscript or its supporting information, or deposited to a public repository. For example, in addition to summary statistics, the data points behind means, medians and variance measures should be available. If there are restrictions on publicly sharing data—e.g. participant privacy or use of data from a third party—those must be specified.

Reviewer #1: Yes

Reviewer #2: Yes

4. Is the manuscript presented in an intelligible fashion and written in standard English?

PLOS ONE does not copyedit accepted manuscripts, so the language in submitted articles must be clear, correct, and unambiguous. Any typographical or grammatical errors should be corrected at revision, so please note any specific errors here.

Reviewer #1: Yes

Reviewer #2: Yes

5. Review Comments to the Author

Please use the space provided to explain your answers to the questions above. You may also include additional comments for the author, including concerns about dual publication, research ethics, or publication ethics. (Please upload your review as an attachment if it exceeds 20,000 characters)

Reviewer #1:

This manuscript "Assessment of a decentralization model in improving treatment and care of visceral leishmaniasis in Turkana County, Kenya: A mixed method study" by Mbui et al. is very interesting. The authors evaluated the impact of the decentralization of VL services in the city of Turkana, Kenya to inform policy at the county and national levels using a mixed methods cross-sectional survey conducted in four health facilities. The authors demonstrated that teaching the communities about the symptoms is essential to reduce delays in looking for reatment. Unfortunately, the authors did not try to replicate the study in another county.

>> The decentralization model was first implemented in Turkana County for a period of 5 years (2018-2022) by an NGO called FIND. Only Turkana County has implemented this model for five years. Therefore, this current study sought to assess the impact of this model in this county in expanding VL services to the marginalized affected communities that are far from the main health facilities. The implementation of the model was conducted independently by FIND, while the authors are simply assessing the effects of the expansion. With these results, the decentralization model can be adopted in other endemic counties in Kenya.

Reviewer #2:

1. Title need to be precise as per research findings.

>> The study assessed several parameters related to the decentralization model. The results of this assessment have been clearly outlined in the results section. The title is focused on the main aim of the study, which was to assess the decentralization model.

2. Abstract should more focused as per the findings not in general information.

>> The abstract has clearly outlined the key findings of this paper under the results subsection. We have slightly, added more key results in this subsection, see lines 39-40.

3. Methodology should be more precise and well described of the survey process.

>> The design of the study followed a secondary data abstraction and analysis process for the quantitative data. The qualitative data was collected using clearly defined data collection guides, which included in-depth interviews (IDIs) and key informant interviews (KIIs). The survey process for both quantitative and qualitative data collection has been clearly described in the methods section.

4. Result should well describe in diagnosis, treatment and follow up of visceral leishmaniasis.

>> The results section clearly describes the number of patient files abstracted (lines 215-221), VL cases diagnosed (subsection 3.3), and patients treated and followed up (subsection 3.4). Further, in the introduction, we have mentioned how VL is diagnosed and treated in Kenya (lines 74-91).

5. Clarity of Figure 1 is very less, it need to be change with a good picture.

>> We have deleted the original Figure 1 and provided another figure, which is now clear. The new figure is, however, showing the locations of the study health facilities.

6. Legends to the figures and tables should be self-explanatory.

>> We have revised the legends of the figures and tables to make them explicit and self-explanatory.

7. The discussion need to be revised to include a comprehensive analysis with critical examination of hypotheses. A more nuanced discussion of the implications of findings for public health and policy would strengthen this section.

>> The study is a descriptive secondary analysis that had no hypotheses stated. The discussion has been well focussed to state the public health and policy implications of the decentralization model, see lines 436-438, 465-467, 493-496, and 508-509.

8. In the Discussion section author should mention about the importance of the study.

>> The importance has been clearly started in lines 436-438.

9. Reference list need to be updated

>> The current references are fairly recent, ranging from 2010 to 2024. Importantly, VL being a neglected disease, not many studies are evaluating VL programmes in East Africa and the larger Africa region.

10. Typological errors need to be corrected throughout the manuscript.

>> We have thoroughly reviewed the manuscript to identify any typographical mistakes. Additionally, we passed the paper to a third independent reviewer for copy-editing.

11. Scientific names should be in proper form throughout the manuscript.

>> We have corrected scientific names throughout the manuscript.

12. The study needs a general revision in terms of language and grammar.

>> We have thoroughly reviewed the manuscript to identify any typographical, language and grammatical mistakes. Additionally, we passed the paper to a third independent reviewer for copy-editing.

6. PLOS authors have the option to publish the peer review history of their article ([what does this mean?](https://journals.plos.org/plosone/s/editorial-and-peer-review-process#loc-peer-review-history)). If published, this will include your full peer review and any attached files.


If you choose “no”, your identity will remain anonymous but your review may still be made public.


**Do you want your identity to be public for this peer review?** For information about this choice, including consent withdrawal, please see our [Privacy Policy](https://www.plos.org/privacy-policy).

Reviewer #1: **Yes:** Rajendranath Ramasawmy

Reviewer #2: No

[NOTE: If reviewer comments were submitted as an attachment file, they will be attached to this email and accessible via the submission site. Please log into your account, locate the manuscript record, and check for the action link "View Attachments". If this link does not appear, there are no attachment files.]

While revising your submission, please upload your figure files to the Preflight Analysis and Conversion Engine (PACE) digital diagnostic tool, <https://pacev2.apexcovantage.com/>. PACE helps ensure that figures meet PLOS requirements. To use PACE, you must first register as a user. Registration is free. Then, login and navigate to the UPLOAD tab, where you will find detailed instructions on how to use the tool. If you encounter any issues or have any questions when using PACE, please email PLOS at <a href="mailto:figures@plos.org">figures@plos.org. Please note that Supporting Information files do not need this step.
