# Supplementary material for: Assessment of a decentralization model in improving treatment and care of visceral leishmaniasis in Turkana County, Kenya: A mixed method study
Source: PLoS One. 2025 May 20;20(5):e0323990. doi: 10.1371/journal.pone.0323990 (PMC12091879; doi:10.1371/journal.pone.0323990)
Supplement: Attachment — Submitted filename: Response_to_reviewers_auresp_2.docx [file pone.0323990.s003.docx]

Review Comments to the Author

Please use the space provided to explain your answers to the questions above. You may also include additional comments for the author, including concerns about dual publication, research ethics, or publication ethics. (Please upload your review as an attachment if it exceeds 20,000 characters)

Reviewer #1: I thank the authors for addressing all my comments. This work is interesting to see how patients perceived the disease and highlight the purpose of educating caregivers to be able to provide rapid care and treatment.

Author response: Thank you for taking your time to review our manuscript and for your valuable comments that improved the presentation of this work.

Reviewer #3: The authors’ responses to the editorial comments are noted. It is particularly noted that the data has been made accessible in the GitHub platform.
In the previous reviews, there were concerns with the rigor and appropriateness of the statistical analysis.

The response to a previous review question on the abstract is unsatisfactory. It might be better to have more details in the methods and results section rather than the background section of the abstract. The authors should consider reviewing the abstract further.

Author response: Thank you for this comment, we have reviewed and revised the abstract appropriately. However, due to the word count restrictions in the abstract section, we were unable to include extensive details.

The response to Reviewer 2’s comment number 7 reveals a concern: In the absence of a hypothesis, it is important to have clearly stated research questions.

Author response: We have added the research questions appropriately. See lines 107-113.

Methods
Study Design: Is there a specific reason for not involving sub-county health management teams in the KIIs?

Author response: Thank you for the question. The study sought to interview key personnel at the county level who influence and implement policy decisions at the county level. This group is the county health management team.

Line 127 to 128: Is there a chance that some cases were counted twice (from stock management forms and from patient records alluded to earlier and outlined in lines 134 - 135)?

Author response: The cases were not counted twice. Each of the four forms served different purposes. For instance, individual demographic and treatment details were captured in the case management form, laboratory diagnosis results for each individual was captured in the laboratory logs, monthly summaries of all individuals diagnosed and treated for VL in the facility were captured in the monthly summary logs, and all VL test kits and medications administered to the patients in a month in the facility were summarized in the monthly stock management log.

Results
In Table 1, it is interesting that the county referral hospital had no cases from 2018 to 2020 then suddenly there were 91 cases in 2021. Any explanation for this steep rise?

Author response: Thank you for pointing this one out. We have replaced zero with missing records (MR). To indicate that the facility did not have VL case management patient records for the specific years under review.

Line 198: When reporting measures of central tendency, it is best to consider which one to report between SD and IQR, based on the value of the SD versus the mean. In this case, the IQR should have been reported because the data looks skewed.

Author response: Despite the skewed nature of the data for some variables, we reported the mean throughout the descriptive analysis because it is more informative and we were not performing any statistical tests other than the descriptive statistics. Further, the range (minimum and maximum), is more informative than the IQR in this context.

Are there specific reasons for not conducting bivariate analysis and multivariable logistic regression analysis were not conducted? How do we assess the statistical significance of your findings?

Author response: As we had stated earlier, the study used descriptive analysis rather than inferential analysis to assess the variables of choice. The study did not have any hypothesis to be tested using the logistic regression models or any other inferential techniques.

A key limitation of your study is that it cannot tell us whether there were other factors at play (potential confounders). For example, in 2020 and 2021, COVID-19 played a major role in health-seeking behavior. In addition, over time, most counties in Kenya have invested in more health facilities.

Author response: Thank you for your comment. However, conforming to the scope of this study, we were not determining factors associated with health seeking behavior or their potential confounders. We have added this as a limitation. See lines 495-497.
